# IgA-Dominant Infection-Related Glomerulonephritis Due to *Mycobacterium avium*: A Case Report

**DOI:** 10.1016/j.xkme.2026.101383

**Published:** 2026-04-30

**Authors:** Ruben Visch, Arjan van Laarhoven, Loek Smits, Dominique van Midden, Anne-Els van de Logt

**Affiliations:** 1 Department of Nephrology, Radboud University Medical Center, Radboud Institute for Health Sciences, Nijmegen, the Netherlands; 2 Department of Internal Medicine and Radboud Community for Infectious Diseases, Radboud University Medical Center, Radboud Institute for Health Sciences, Nijmegen, the Netherlands; 3 Department of Pathology, Radboud University Medical Center, Radboud Institute for Health Sciences, Nijmegen, the Netherlands

**Keywords:** Glomerulonephritis, IgA-dominant postinfectious glomerulonephritis infection, infection, *Mycobacterium avium*

## Abstract

Immunoglobulin A-dominant infection-related glomerulonephritis (IgA-DIRGN) is a rare variant of acute postinfectious glomerulonephritis, typically seen in elderly individuals with comorbid conditions. IgA-DIRGN is characterized by diffuse endocapillary proliferation and the formation of mesangial deposits of IgA and C3, leading to inflammation and subsequently resulting in acute kidney injury, proteinuria, hematuria, and hypocomplementemia. In this report, we describe an immunocompromised 68-year-old man who developed acute kidney injury, nephrotic-range proteinuria, and microscopic hematuria following a splenic *Mycobacterium avium* abscess. Kidney biopsy showed endocapillary proliferative glomerulonephritis with IgA and C3-dominant granular deposits, consistent with IgA-DIRGN. Progression of kidney dysfunction during antimycobacterial therapy prompted the use of glucocorticoids 1 month after the initial presentation and eventually a splenectomy 1 month later, followed by renal recovery. To our knowledge, this is the first case to report the association between a nontuberculous mycobacterial infection and IgA-DIRGN, emphasizing the importance of source control and illustrating the usefulness of steroids in this patient.

## Introduction

Immunoglobulin A-dominant infection-related glomerulonephritis (IgA-DIRGN) is a rare morphological variant of acute postinfectious glomerulonephritis (GN). Unlike the classical form of postinfectious GN, which is typically associated with streptococcal infections in children,^1^ IgA-DIRGN predominantly affects elderly adults with comorbid conditions such as diabetes or chronic kidney disease. It is commonly linked to skin infections and visceral abscesses, particularly those caused by *Staphylococcus aureus*, and typically presents with acute kidney injury, proteinuria, hematuria, and hypocomplementemia.^2^ Histologically, in 63%-80% of patients it manifests with diffuse endocapillary proliferative and exudative GN with prominent neutrophil infiltration.^3^^,^^4^ A minority may have isolated mesangial proliferative or crescentic GN. Immunofluorescence shows mesangial staining in a codominant pattern with IgA and C3. Electron microscopy demonstrates electron-dense deposits in the mesangium and in the capillary walls.^4^

Infection-related glomerulonephritis (IRGN) because of nontuberculous mycobacteria and lepra is exceedingly rare and has only been described in the literature in a few case reports.^5^, ^6^, ^7^, ^8^, ^9^ All of these cases involved individuals aged ≥60 years infected with slow-growing mycobacteria. Of the 5 cases, 2 were pulmonary (*Mycobacterium avium*) and 3 extrapulmonary, infecting the skin and soft tissues (*M. avium*, *M*. *gordonea*, and *M*. *leprae*). *M*. *avium* is an environmental, slow-growing nontuberculous mycobacteria that typically affects immunocompromised individuals, causing pulmonary disease, disseminated infections, or localized abscesses.^10^ Although renal involvement of *M. avium* infections is extremely uncommon, it may trigger immune-mediated glomerular disease in predisposed individuals.

To our knowledge, IgA-DIRGN because of *M*. *avium* has not been described in the literature.

## Case Report

**Figure 1:**
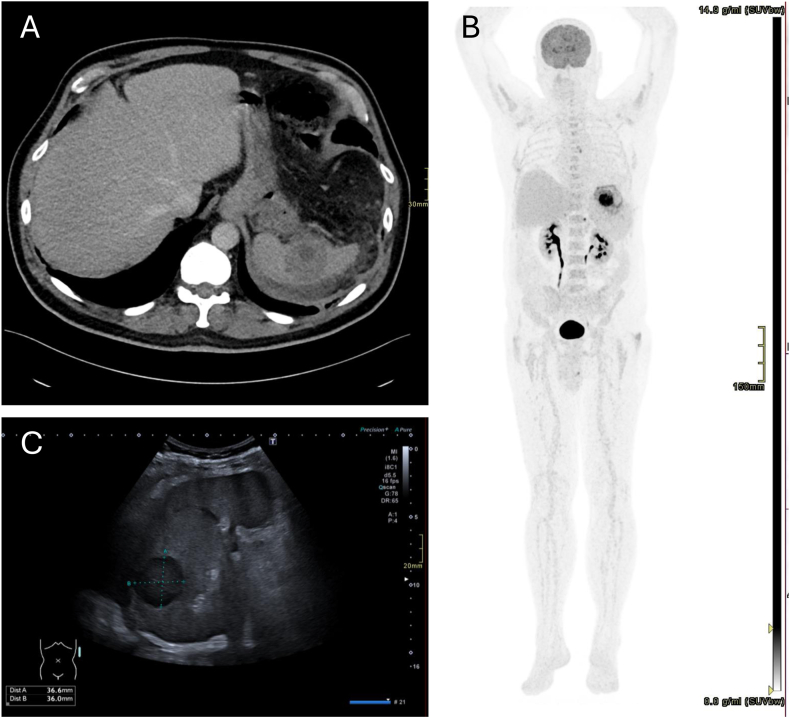
(A) Computed tomography revealing a large splenic abscess, soft tissue window, axial view. (B) Positron emission tomography ruled out metastatic infections, standardized uptake value, 0-15 g/mL. (C) Ultrasound of splenic abscess.

A 68-year-old man with stage II sarcoidosis and rheumatoid arthritis, treated with methotrexate and adalimumab, presented with a 2-week history of fever, night sweats, 6 kg weight loss, and nausea. He has also been experiencing hiccups over the past few days. A computed tomography scan showed a large splenic abscess, and positron emission tomography ruled out metastatic infections. The abscess measured 37 × 36 × 37 mm on ultrasound (Fig 1). Immunosuppressive medications were discontinued, and the hiccups were treated with baclofen and metoclopramide. A percutaneously acquired culture of the abscess grew *M*. *avium*, prompting initiation of azithromycin, rifampicin, and ethambutol.

Two weeks later, he was readmitted because of systemic inflammation and the progression of the splenic abscess, which had increased in size to 56 × 49 × 42 mm despite antimycobacterial therapy. In addition, serum creatinine level rose from 0.80 mg/dL at the time of the initial admission to 1.32 mg/dL on readmission. Urinalysis showed >400 red blood cells per field of view (>50% dysmorphic) with acanthocytes and red blood cell casts, and 5.8 g/24 h of proteinuria. Complement levels showed normal C3 (956 mg/L) and C4 (293 mg/L) levels. Antinuclear antibodies, antimyeloperoxidase antibodies, antiproteinase 3 antibodies, and glomerular basement membrane antibody were negative.

As serum creatinine level increased to 1.90 mg/dL, oral prednisolone (1 mg/kg, 80 mg) was initiated because of suspicion of IRGN.

**Figure 2:**
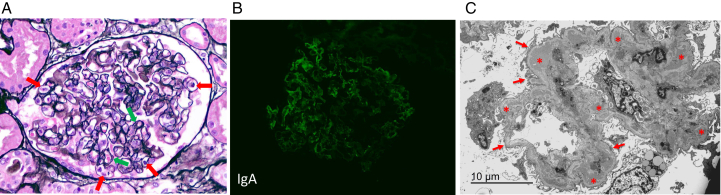
(A) Light microscopy (hematoxylin and eosin, original magnification, ×400) showing a glomerulus with endocapillary proliferation (red arrows) and double contours (green arrows). (B) Immunofluorescence demonstrated dominant IgA (2+) and C3 (2+) staining, with trace IgG, IgM, and C1q. Light chain staining was polyclonal (kappa 1+, lambda 1+). Staining showed a predominantly granular pattern involving both the glomerular mesangium and capillary walls. In addition, focal hyaline thrombus-like intracapillary deposits were present. Trace IgG was observed segmentally in a distribution overlapping with the dominant deposits, whereas IgM and C1q showed only weak and irregular staining. (C) Electron microscopy showed widespread podocyte foot process effacement (arrows). Electron-dense immune deposits were identified in the mesangium and subendothelium (∗). Associated subendothelial lucent zones reflected endothelial injury/activation. No subepithelial hump-like deposits, organized deposits, or tubuloreticular inclusions were identified.

To establish the underlying diagnosis, a kidney biopsy was performed. Twenty-two glomeruli were identified, none globally sclerosed. The glomeruli demonstrated a diffuse mild endocapillary proliferative pattern with focal double-contour formation and hyaline pseudothrombi. Erythrocyte casts were present in multiple tubules. Immunofluorescence demonstrated dominant IgA (2+) and C3 (2+) with trace IgG, IgM, and C1q. Granular deposits were predominantly capillary wall-associated and polyclonal. Electron microscopy showed diffuse foot process effacement with mesangial and subendothelial immune deposits (Fig 2).

After 1 week of prednisolone, serum creatinine showed mild improvement, and oral glucocorticoids were tapered after transgastric drainage of the splenic abscess. Unfortunately, source control remained inadequate, as the patient continued to experience fever and rising C-reactive protein; over the following days, serum creatinine level increased to 3.09 mg/dL. To reduce the antigenic load, a splenectomy was performed. Eleven days later, serum creatinine level had decreased to 1.82 mg/dL, and the patient was discharged.

Two weeks later, the patient was readmitted to the hospital because of increasing inflammation and progressive renal insufficiency. A computed tomography scan showed increased ascites, enlargement of the known abscesses in the splenic lodge, and new abscess formation, consistent with progression of the *M*. *avium* infection. However, during this admission, the patient chose to leave the hospital without further diagnostic workup. Therefore, oral glucocorticoids were increased, and he was discharged.

Two months later, the patient was readmitted with fever and an abscess with intestinal flora at the site of the previous splenic bed, attributed to fistula formation from the stomach after transgastric drainage. *M. avium* was no longer identified in the abscess by culture or polymerase chain reaction, and his kidney function remained stable throughout this admission with serum creatinine level decreasing to 1.28 mg/dL and further improving to 1.13 mg/dL at the end of antimycobacterial therapy 5 months later.

## Discussion

Here, we describe the rare manifestation of an IgA-DIRGN in an immunocompromised 68-year-old man with *M*. *avium* splenic abscess. The patient presented with rapidly progressive kidney injury, nephrotic-range proteinuria, and microscopic hematuria with dysmorphic red blood cells and casts, and biopsy findings of endocapillary proliferative GN with IgA and C3-dominant granular deposits consistent with IgA-DIRGN. The presence of glomerular basement membrane double contours suggests chronic endothelial injury, which may have been exacerbated by ongoing infection and inflammation.

At least 3 of the following 5 criteria should be fulfilled to diagnose an IRGN, as proposed by Nasr et al^11^: (1) clinical or laboratory evidence of infection preceding or at the onset of GN; (2) depressed serum complement; (3) endocapillary proliferative and exudative GN; (4) C3-dominant or codominant glomerular immunofluorescence staining; or (5) ‘hump-shaped’ subepithelial deposits on electron microscopy. IRGN is classically thought to be driven by host immune responses to specific pathogens, triggering a polyclonal increase in serum IgA level, massive T-cell activation, and lymphokine release. This process may be facilitated by bacterial superantigens, which are unconventional antigen proteins that bind immune receptors outside their usual recognition sites.^12^^,^^13^

**Figure 3:**
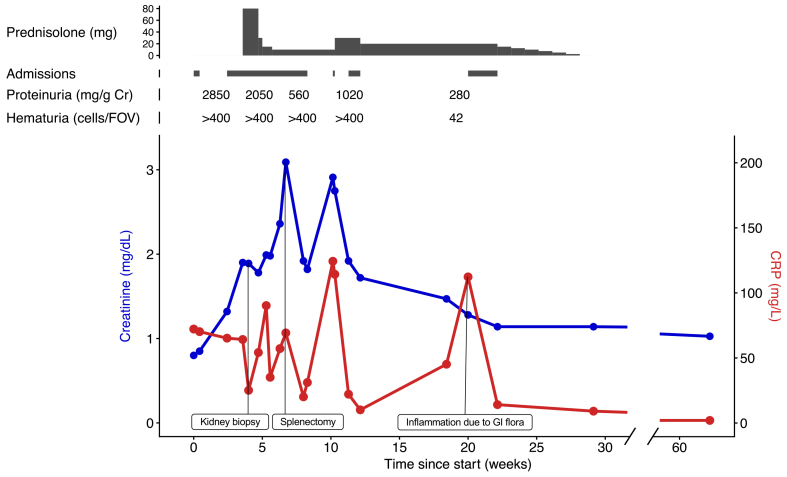
Clinical course and prednisolone treatment. Cr, creatinine; CRP, C-reactive protein; FOV, field of view; GI, gastrointestinal.

To our knowledge, the association of *M*. *avium* with IRGN has only been described in 3 cases (Table 1^5^^,^^6^^,^^9^). To our knowledge, this is the first report of an IgA-DIRGN associated with *M*. *avium*. In contrast to typical acute postinfectious GN, IgA-DIRGN typically occurs during infection.^2^ The prognosis is unfavorable, as 15%-31% of patients progress to end-stage kidney disease.^2^^,^^14^^,^^15^ Treatment of IgA-DIRGN is to treat the underlying infection, while the effectiveness of immunosuppression is controversial and not recommended by Kidney Disease: Improving Global Outcomes (KDIGO) 2021.^16^ In this case, however, because of the declining kidney function and that no prompt therapeutic effect was expected from treatment of the mycobacterial infection (because of difficult source control and the prolonged need for antimicrobial therapy), corticosteroids were initiated. This was followed by only a mild improvement of kidney function. We hypothesize that the large remaining mycobacterial load in the splenic abscess and possible concurrent antigenemia led to immune complex deposition for which splenectomy was the resolution. Fig 3 shows the clear correlation between an increase in C-reactive protein and a increase in serum creatinine level. Notably, during the hospitalization, in which gastrointestinal flora was cultured from an abscess in the splenic lodge months after the splenectomy, there was no deterioration in kidney function despite an increase in C-reactive protein. This supports the hypothesis that the decline in kidney function is because of *M*. *avium-*induced inflammation.

**Table 1:** Clinical, Laboratory, and Renal Morphology On Presentation in Patients With a *Mycobacterium avium* Infection and Infection-related Glomerulonephritis

Case No.	Reference	Age (y)	Sex	Proteinuria	Hematuria	Creatinine (mg/dL)	Kidney Biopsy		Outcome
							LM	IF Antibodies	
1	Chen et al^5^ (2007)	72	F	1.9 g/24 h	+++	2.2	Proliferative GN, crescents	IgG 2+ IgM 1+ IgA −	Recovery without additional therapy
2	Huang et al^6^ (2023)	73	M	>300 mg/g	++	2.1	Proliferative GN, crescents	IgG 3+ IgM 1+ IgA −	Died
3	Indhumathi et al^7^ (2020)	73	F	1.5 g/24 h	20-30 cells/HPF	0.8	Proliferative GN, crescents	IgG 3+ IgM 1+ IgA −	Recovery with steroids
4	Present case	68	M	5.8 g/24 h	+++	0.8	Proliferative GN	IgG trace IgM trace IgA 2+	Recovery with steroids

Abbreviations: GN, glomerulonephritis; HPF, high power field; Ig, immunoglobulin; IF, immunofluorescence; LM, light microscopy.

Although extrapulmonary *M*. *avium* typically affects immunocompromised hosts, kidney injury is very uncommon and has not been well characterized. Our patient showed a favorable prognosis when steroids were started, as further renal deterioration was prevented. This case also highlights the crucial role of source control in managing IRGN, as ongoing antigenic stimulation may drive disease progression despite assumed appropriate antimycobacterial therapy. Further studies are needed to better understand the pathophysiology, optimal treatment strategies, and long-term outcomes of IgA-DIRGN.
